# Mesenchymal Stem Cell Therapy for Acute Myocardial Infarction: Protocol for a Systematic Review and Meta-Analysis

**DOI:** 10.2196/60591

**Published:** 2025-02-06

**Authors:** Michael Vincent DiCaro, Brianna Yee, KaChon Lei, Kavita Batra, Buddhadeb Dawn

**Affiliations:** 1 Division of Cardiovascular Medicine Department of Internal Medicine Kirk Kerkorian School of Medicine at UNLV Las Vegas, NV United States

**Keywords:** mesenchymal stem cells, mesenchymal stromal cells, progenitor cells, acute myocardial infarction, outcomes, stem cell, myocardial, protocol, systematic review, meta-analysis, medical therapy, therapy, cardiac, efficacy

## Abstract

**Background:**

Medical therapy and interventional approaches have improved outcomes in patients with acute myocardial infarction (MI). However, these strategies are inadequate for replacing cells lost during tissue ischemia, thereby leaving behind noncontractile scar tissue. The anti-inflammatory and immune modulating properties of mesenchymal stem cells (MSCs) may prove useful in inducing functional cardiac regeneration following acute MI.

**Objective:**

This is a protocol for systematic review and meta-analysis that will aggregate and synthesize high-level clinical data on the effects of MSC therapy for acute MI. The findings of this study may serve as evidence for clinicians and researchers in guiding the use of MSC therapy as an adjunct to reperfusion and optimal medical therapy in patients with acute MI.

**Methods:**

The proposed systematic review is registered with PROSPERO (International Prospective Register of Systematic Reviews). A systematic search of bibliographical databases, including Embase, PubMed, and Cochrane was conducted from inception to June 2023 to identify English-language human studies with adult patients receiving MSC therapy and optimal medical therapy for acute MI in comparison with respective controls. Article screening was performed using PRISMA (Preferred Reporting Items for Systematic Reviews and Meta-Analyses) guidelines. Data on functional cardiac outcomes and major adverse cardiac events were extracted and analyzed as primary outcomes.

**Results:**

Literature search and article screening commenced in June 2023. Data extraction and analysis will be completed by October 2024. The findings will be synthesized and reported by the end of November 2024.

**Conclusions:**

This systematic review and meta-analysis will summarize the best available updated evidence from published randomized controlled trials on the effects of MSC therapy for the treatment of acute MI. The findings of this systematic review and meta-analysis may shed light on the efficacy of MSC therapy in improving cardiac functional and structural parameters and reducing adverse cardiac events following acute MI.

**Trial Registration:**

PROSPERO CRD42024522398; https://www.crd.york.ac.uk/prospero/display_record.php?RecordID=522398

**International Registered Report Identifier (IRRID):**

DERR1-10.2196/60591

## Introduction

### Background

Acute myocardial infarction (MI) remains a significant cause of morbidity and mortality globally, with nearly 3 million people experiencing an acute MI annually worldwide [1]. During acute MI, blood flow to the myocardium is reduced, resulting in tissue hypoxia, ischemia, and eventual cell death, which in turn, may result in adverse consequences, including left ventricular (LV) dysfunction, heart failure, arrhythmias, cardiogenic shock, and death. Myocardial reperfusion with percutaneous coronary intervention is a standard treatment for acute MI. Time to reperfusion is directly correlated with prognosis; therefore, prompt revascularization should be pursued [2]. After reperfusion, patients receive optimal medical therapy, which has significantly improved outcomes since its advent [3]. However, reperfusion and medications are unable to replenish necrotic cardiac myocytes, and many patients still experience significant morbidity and mortality following acute MI [4]. Following significant tissue infarction, large areas of the myocardium are scarred and rendered nonfunctional, leading to the adoption of regenerative therapies as a possible solution.

Accordingly, regenerative therapies that aim to restore functional cardiac tissue continue to be a topic of clinical research interest. Recent evidence suggests that stem cells may be useful as a method of repairing damaged myocardial tissue [5,6]. In animal models, studies have shown the potential of Mesenchymal stem cells (MSCs) to differentiate into cardiac myocytes, to participate in regenerative signaling through immunomodulation, and paracrine actions [7-9]. So far, human clinical trials have yielded mixed results. Several randomized control trials (RCTs) in patients with acute MI have demonstrated improvement in LV ejection fraction (LVEF), remodeling, myocardial viability, and reduction in hospitalization rates following treatment with MSCs [10-13]. Conversely, other RCTs have shown no difference in LVEF between MSC-treated patients and the standard-of-care [14,15]. Concerns for safety has arisen with the novelty of regenerative therapy, and the evaluation of major adverse cardiac events (MACE) has been the hallmark for safety [10-15]. Particularly, mortality rates (periprocedurally and long-term), malignant arrhythmias, recurrent MI, cerebral vascular accident, and revascularization are among the most common evaluated adverse events [10-15]. Although with no significant difference was noted in previous RCTs, our meta-analysis aims to evaluate the safety of MSC therapy through the evaluation of MACE. There may be added benefit to decreasing MACE outcomes through the use of MSC therapy, which will also be evaluated.

While many clinical trials have examined the efficacy of MSCs in acute MI treatment, the data collection methods, timing of MSC administration, route of MSC administration, and evaluated end points have been heterogeneous. Several articles representing long-term follow-up of original studies have recently been published, which collectively provide additional insights. Aggregation and analysis of the updated data are needed to gain a better understanding of the effects of MSC administration in patients with acute MI.

### Objectives

Several previous meta-analyses have been published on the outcomes of stem cell therapy for acute MI, however, these were based on studies with a heterogeneous mixture of stem cells, not exclusively MSCs. In addition, the existing meta-analyses included only original RCTs without the follow-up studies, thereby obscuring insights related to longer-term outcomes. The current systematic review aims to provide the most comprehensive and updated evidence regarding MSC transplantation solely for the treatment of acute MI, along with a meta-analysis focused specifically on RCTs, including follow-up RCTs to evaluate long-term outcomes of MSC therapy. The meta-analysis will compare the effects of MSC injection and standard therapy on LVEF, LV end-systolic volume (LVESV), LV end-diastolic volume (LVEDV), and MACE as primary end points. Secondary end points will include, but not limited to, myocardial viability, myocardial perfusion defect, and stroke volume.

## Methods

### Review Question

This study aimed to answer the following question: “What are the short-term and long-term effects of MSC therapy in patients with acute MI?”

### Eligibility Criteria

Inclusion and exclusion criteria were initially determined using the PECOS (Population, Exposure, Comparator, Outcome, Study Design) framework. Detailed PECOS criteria for the meta-analysis are denoted in Multimedia Appendix 1.

The initial systematic review included RCTs, single-arm studies, and case series. The meta-analysis included RCTs and secondary reports based on the original studies. English-only original articles published before June 15, 2023, were included; case reports, abstract-only articles, systematic reviews, meta-analyses, animal studies, commentaries, position papers, opinions, and editorials were excluded from the meta-analysis. Studies enrolling adult patients aged 18 years and older with acute MI who received MSC therapy within 1 month of MI were included. Studies using MSCs, mesenchymal stromal cells, and mesenchymal progenitor cells as therapeutic substrates were included. Studies that included patients with recurrent MI in their study were excluded. Studies with other types of stem cells or combinatorial cellular mixtures were excluded. Studies that did not report the outcomes of interest were excluded.

### Information Sources and Search Strategy

Bibliographic databases, including PubMed, Cochrane, and Embase were queried from inception to June 2023. The search strategy was guided by 2 librarian experts in medical sciences and was reviewed and devised in accordance with the Peer-Reviewed Electronic Search Strategy guidelines [16].

The original search strategy was formulated for PubMed and used PubMed syntax. Additional searches were modified and optimized for subsequent databases. Given the specificity of the question at hand, the search strategy used key search terms without the use of MeSH (Medical Subject Headings).

The full search strategy, including the development of the final search terms, can be found in Table S1 in the Multimedia Appendix 2. Keywords in the final search strategy included the following: mesenchymal stem cell, progenitor cell, mesenchymal stromal cell, ST-elevation MI, ST-elevation myocardial infarction, and acute MI.

### Selection Process

All retrieved articles were reviewed by 2 independent investigators to check for inclusion and exclusion criteria in a stepwise process to include title screening, abstract screening, and full-text screening. Reasons for the exclusion of articles will be addressed in greater detail in the final publication.

### Study Records

#### Data Collection Process and Management

Data extraction from the eligible full-text articles was performed by 2 investigators who independently extracted the data elements of interest to ensure accuracy and completeness. Extracted data and records are maintained in separate Microsoft Excel sheets by the 2 investigators, as well as a combined Microsoft Excel sheet after consensus.

Incongruencies in data extraction were resolved through discussion with a third senior investigator to achieve consensus.

#### Data Items/Elements

Data extraction elements for both systematic review and meta-analysis are outlined in Table S2, which can be found in Multimedia Appendix 3.

Information that required further clarification and/or required additional data were attempted to be resolved by contacting the corresponding authors of the respective articles; queries that yielded no response were not included in the data extraction or analysis.

#### Outcomes

The primary outcomes of the meta-analysis included (1) effects of transplantation on LVEF, (2) LVESV, (3) LVEDV, and (4) MACE.

The secondary outcomes that included (1) myocardial viability, (2) myocardial perfusion defect index, (3) coronary flow reserve, (4) adenosine-induced minimal vascular resistance index, and (5) stroke volume.

### Quality Analysis

#### Overview

Quality assessments for strength of the body of evidence of all included studies will be performed using the National Heart, Lung, and Blood Institute (NHLBI) quality assessment tool. A total of 2 investigators will independently screen full texts for quality. Scoring will be performed independently. All essential components of original research studies will be evaluated using the NHLBI scoring checklist (Multimedia Appendix 4). Study quality will model the scoring protocol performed by Elks et al [17].

#### Risk of Bias Assessment

**Figure 1:**
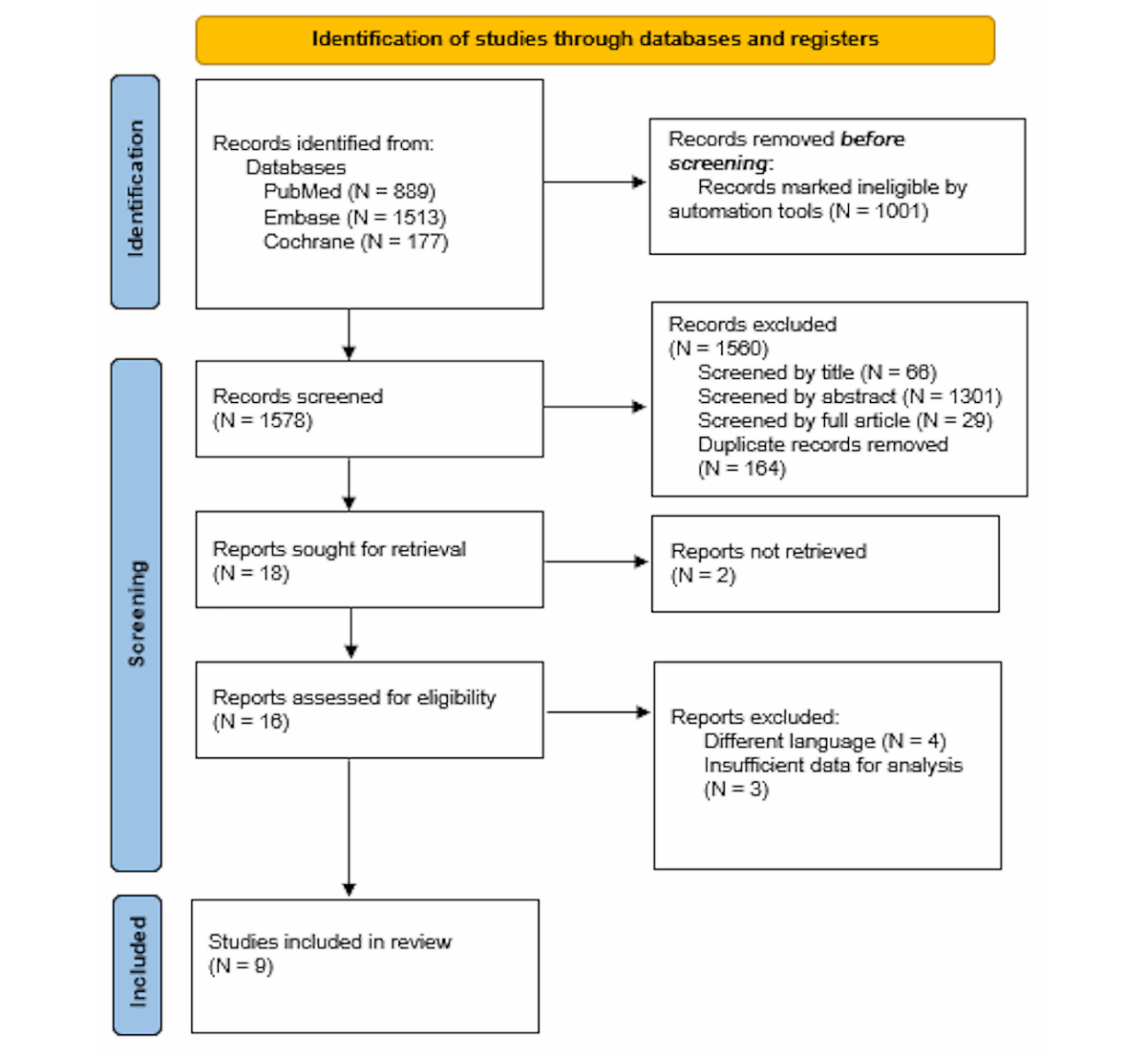
The PRISMA (Preferred Reporting Items for Systematic Reviews and Meta-Analyses) flow diagram modified for the current study selection process.

Within the quality assessment, articles selected for analysis underwent assessment for risk of bias. Bias was assessed by examining randomization and adequacy of randomization, blinding, and attrition. Specific questions are outlined in Figure 1 below. Meta-biases will be assessed as appropriate.

### Data Synthesis

First results of all finally included studies will be described succinctly in the form of a summary table. A random-effects model will be used to calculate pooled estimates as this is a more robust estimate regardless of heterogeneity [18]. Cochran Q and *I^2^* statistics will be used as indicators of heterogeneity. The pooled estimates of the primary end points (eg, LVEF, LVESV, and LVEDV) will be calculated as the weighted mean differences with 95% CIs using the Comprehensive Meta-analysis Package (CMA version 3.0). For dichotomous outcomes, Peto odds ratios will be used as this allows the inclusion of the continuity correction of 0.5 to all 0 cells outcomes and accounts for the expected rarity of events. Sensitivity analysis will be conducted to identify studies that may severely affect the pooled estimates. The Exploratory subgroup analyses by different moderator variables (eg, MSC source, MSC route of administration, location of MI, and duration of follow-up, etc) will also be conducted to examine sources of heterogeneity. A funnel plot and Egger linear regression test will be used to assess publication bias [19]. The significant level will be set as 2-sided and *P*<.05. Forest plots will be used to present the data.

### Data Analysis and Presentation

Data will be presented in a tabular form and as a conclusive summary of our analysis. A data extraction form will be developed and agreed upon by all investigators before article analysis occurs. Selected articles will be present in the rows of the table, and variables will be present in the columns of the table. Quantitative analysis of all extracted variables will be performed.

### Ethical Considerations

**Figure 2:**
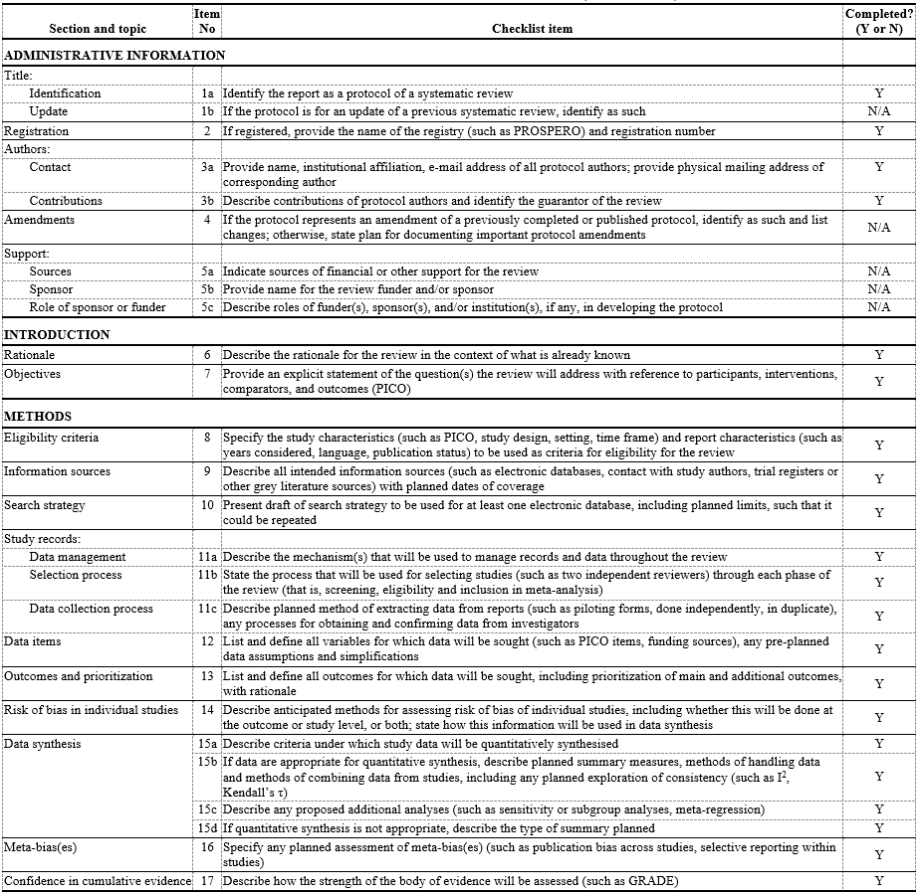
PRISMA-P (Preferred Reporting Items for Systematic Reviews and Meta-Analyses) checklist completed for the current study.

IRB or institutional review was not needed in this systematic review and meta-analysis since this design relies only on the findings from the previously published data and did not involve direct interaction with human participants. However, to adhere to a strict methodology, the protocol of this systematic review and meta-analysis was registered with PROSPERO (International Prospective Register of Systematic Reviews) registration number CRD42024522398. PROSPERO is an international database of prospectively registered systematic reviews, which provides a unique permanent registration number to the protocol that prevents duplication, thereby reducing reporting bias. In addition, PRISMA-P (Preferred Reporting Items for Systematic Reviews and Meta-Analyses Protocols) checklist was completed during the process to ensure comprehensive and quality results (Figure 2).

## Results

The development of the search strategy for this systematic review and meta-analysis began in June 2023. The search strategy underwent peer-reviewed electronic search strategy review with 2 academic librarians in June 2023. The final search strategy was agreed upon by all investigators in July 2023. Databases were queried and articles were screened between September 2023 and October 2023. Data extraction took place between November 2023 and February 2024. Data analysis is currently taking place as of March 2024 with the final analysis anticipated to be completed in October 2024. It is anticipated that findings will be synthesized and reported by the end of November 2024. Findings will be organized, summarized, and submitted for publication in a peer-reviewed journal. The goal of this work is to provide a comprehensive analysis and enhance the understanding of the safety and effectiveness of MSC therapy in acute MI. A timeline is provided in Table 1.

Preliminary searches performed in PubMed, Embase, and Cochrane databases yielded 1578 articles after appropriate filters were applied. A total of 889 articles were returned from PubMed, of which 521 were human studies. A total of 1,513 articles were returned from Embase, of which 880 were human studies. A total of 177 articles were returned from the Cochrane database, which were all reviews of human studies (Figure 1). A detailed step-by-step outline of our search strategy, including search terms, limit criteria, and results is provided in Table S1 of the Multimedia Appendix 2 (adapted from Page et al [20]).

**Table 1:** Timeline of project task completion.

Task	Jun 2023	Jul 2023	Aug 2023	Sep 2023	Oct 2023	Nov 2023	Dec 2023	Jan 2024	Feb 2024	Mar 2024	Apr 2024	May 2024	Jun 2024	Jul 2024	Aug 2024	Sep 2024	Oct 2024	Nov 2024
Design search strategy	✔	✔																
Title screening			✔															
Abstract screening				✔														
Full-text screening				✔	✔													
Data extraction						✔	✔	✔	✔									
Synthesis and risk of bias assessment										✔	✔							
Data analysis										✔	✔	✔	✔	✔	✔	✔	✔	
Abstract and manuscript drafting												✔	✔	✔	✔	✔	✔	✔
Data dissemination																		✔

## Discussion

### Overview

This systematic review and meta-analysis will be a comprehensive review and analysis of the existing relevant literature pertaining to the efficacy of MSC therapy in patients enduring from acute MI. We anticipate that the current findings will be congruent with the previous observations regarding the outcomes and safety of MSC therapy. We also aim to glean insights with regard to changes in LVEF, LVESV, and LVEDV as a result of MSC therapy. In addition, we will examine the pooled event ratio estimates of MACE, including death, recurrent MI, need for revascularization, and stroke, compared with control, to draw conclusions on the safety of MSC therapy.

In an attempt to ensure the comprehensiveness of the assessment of relevant data, a narrative synthesis of studies that lie at relatively lower levels of hierarchical evidence as compared with the gold standard (RCTs) was also performed. These additional qualitative findings allow us to gain a complete understanding of several factors, including timing and route of administration, source of cells, and other variables that may impact the outcomes of MSC therapy.

### Significance

We anticipate that our results, by virtue of including data from recently published RCTs, will significantly advance the existing evidence regarding the safety and efficacy of MSC therapy. Previous meta-analyses examining the effects of MSC therapy in ischemic heart diseases often combined with chronic and acute ischemic heart diseases [21,22], whereas we plan to focus on acute MI therapy alone. In addition, previous meta-analyses reviewed a variety of regenerative cells [6] for the treatment of acute MI, whereas our focus specifically on mesenchymal stem and progenitor cells will allow us to comment on the efficacy of a specific cell type in the management of acute MI. Finally, while our meta-analysis examines primary RCTs, we have also included secondary/follow-up RCTs of original studies in our analysis, which will provide additional information regarding the longer-term and additional end points for subgroup analysis.

### Limitations

This meta-analysis will have several limitations. First, there will be inherent heterogeneity among included studies due to the variability of research methodologies undertaken by each unique study. We anticipate heterogeneity in several variables, including the timing and route of MSC administration, the tissue source of MSCs, the methods used to measure primary and secondary end points (for example, echocardiogram vs angiography vs cardiac magnetic resonance imaging), and the follow-up period after MSC transplantation. Second, there is a risk that relevant literature will be missed because studies with insignificant findings or low sample size are seldom published, thus leading to publication bias. Finally, there will be inherent reviewer bias when selecting articles during the screening process, which will be mitigated by using an individualized screening process with 2 independent reviewers, along with a third senior reviewer to resolve disagreements on article selection.

### Dissemination Plan

The findings of this systematic review and meta-analysis will be submitted as a manuscript to a peer-reviewed journal by the end of 2024. Derivations of this work will be submitted as abstracts to academic conferences.

### Conclusions

This systematic review and meta-analysis will provide a comprehensive review of the safety and efficacy of MSC therapy in patients with acute MI. It will include an analysis of data on clinically relevant primary end points pertaining to functional cardiac parameters, such as LVEF, LVESV, and LVEDV. It will also examine specific safety outcomes by performing a subgroup analysis on MACE. This review will also provide qualitative synthesis of evidence related to MSC therapy, which will generate additional insights toward its potential future clinical applications in a broader spectrum of cardiovascular pathologies.

## Appendix

Multimedia Appendix 1 The PECOS (P-Population; E/I-Exposure/Intervention; C-Comparator; O-Outcome; S-Study design) framework for the eligibility criteria.

Multimedia Appendix 2 Full search strategy for utilized databases.

Multimedia Appendix 3 Elements included in data extraction for meta-analysis purposes.

Multimedia Appendix 4 National Heart, Lung, and Blood Institute scoring checklist.

## Abbreviations

LV: left ventricle
LVEDV: left ventricular end-diastolic volume
LVEF: left ventricular ejection fraction
LVESV: left ventricular end-systolic volume
MACE: major adverse cardiac events
MeSH: Medical Subject Headings
MI: myocardial infarction
MSC: mesenchymal stem cells
NHLBI: National Heart, Lung, and Blood Institute
PECOS: Population, Exposure, Comparator, Outcome, Study Design
PRISMA-P: Preferred Reporting Items for Systematic Reviews and Meta-Analyses Protocols
PROSPERO: International Prospective Register of Systematic Reviews
RCT: randomized control trial

## Data Availability

The datasets generated during and/or analyzed during this study are available from the corresponding author on reasonable request.
