# Supplementary figures and images for: Mesenchymal Stem Cell Therapy for Acute Myocardial Infarction: Protocol for a Systematic Review and Meta-Analysis
Source: JMIR Res Protoc. 2025 Feb 6;14:e60591. doi: 10.2196/60591 (PMC11843057; doi:10.2196/60591)

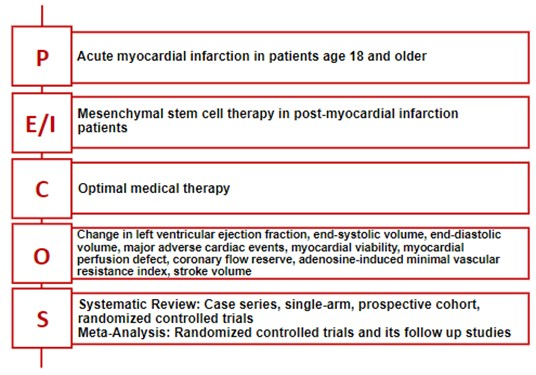

Supplement: Multimedia Appendix 1 [file resprot_v14i1e60591_app1.png]
