# Supplementary material for: Mesenchymal Stem Cell Therapy for Acute Myocardial Infarction: Protocol for a Systematic Review and Meta-Analysis
Source: JMIR Res Protoc. 2025 Feb 6;14:e60591. doi: 10.2196/60591 (PMC11843057; doi:10.2196/60591)
Supplement: Multimedia Appendix 2 [file resprot_v14i1e60591_app2.docx]

| **Database** | **Query used** | **Limit criteria** | **Results** | **Totals** |
| --- | --- | --- | --- | --- |
| **PubMed** |  |  |  |  |
| #1 | “Mesenchymal stem cell*” | Inception – June 2023 | 79,546 |  |
| #2 | “Mesenchymal stromal cell*” | Inception – June 2023 | 10,661 |  |
| #3 | “Progenitor cell*” | Inception – June 2023 | 74,877 |  |
| #4 | #1 OR #2 OR #3 | Inception – June 2023 | 152,137 |  |
| #5 | “ST-Elevation myocardial infarction*” | Inception – June 2023 | 16,956 |  |
| #6 | “STEMI*” | Inception – June 2023 | 16,184 |  |
| #7 | “Acute myocardial infarction*” | Inception – June 2023 | 68,336 |  |
| #8 | #5 OR #6 OR #7 | Inception – June 2023 | 86,304 |  |
| #9 | #4 AND #8 | Inception – June 2023 | **889** |  |
| #10 | #4 AND #8 | Inception – June 2023;  Human Filter | **521** | **521** |
|  |  |  |  |  |
| **Embase** |  |  |  |  |
| #1 | 'Mesenchymal stem cell*' | Inception – June 2023 | 108,377 |  |
| #2 | “Mesenchymal stromal cell*” | Inception – June 2023 | 15,982 |  |
| #3 | “Progenitor cell*” | Inception – June 2023 | 114,722 |  |
| #4 | #1 OR #2 OR #3 | Inception – June 2023 | 225,215 |  |
| #5 | “ST-Elevation myocardial infarction*” | Inception – June 2023 | 22,262 |  |
| #6 | “STEMI*” | Inception – June 2023 | 38,280 |  |
| #7 | “Acute myocardial infarction*” | Inception – June 2023 | 100,206 |  |
| #8 | #5 OR #6 OR #7 | Inception – June 2023 | 136,385 |  |
| #9 | #4 AND #8 | Inception – June 2023 | **1513** |  |
| #10 | #4 AND #8 | Inception – June 2023;  Human Filter | **880** | **880** |
|  |  |  |  |  |
| **Cochrane Library** |  |  |  |  |
| #1 | "Mesenchymal stem cell*" | All Text; Inception – June 2023 | 2157 |  |
| #2 | “Mesenchymal stromal cell*” | All Text; Inception – June 2023 | 415 |  |
| #3 | “Progenitor cell*” | All Text; Inception – June 2023 | 2124 |  |
| #4 | #1 OR #2 OR #3 | All Text; Inception – June 2023 | 4430 |  |
| #5 | “ST Elevation myocardial infarction*” | All Text; Inception – June 2023 | 3209 |  |
| #6 | “STEMI*” | All Text; Inception – June 2023 | 4157 |  |
| #7 | “Acute myocardial infarction*” | All Text; Inception – June 2023 | 10,009 |  |
| #8 | #5 OR #6 OR #7 | All Text; Inception – June 2023 | 13952 |  |
| #9 | #4 AND #8 | All Text; Inception – June 2023 | **177** | **177** |
|  |  |  | **GRAND TOTAL** | **1578** |
|  |  |  |  |  |
