# Supplementary material for: Mesenchymal Stem Cell Therapy for Acute Myocardial Infarction: Protocol for a Systematic Review and Meta-Analysis
Source: JMIR Res Protoc. 2025 Feb 6;14:e60591. doi: 10.2196/60591 (PMC11843057; doi:10.2196/60591)
Supplement: Multimedia Appendix 3 [file resprot_v14i1e60591_app3.docx]

| Study Characteristics | Primary Outcomes | Secondary Outcomes |
| --- | --- | --- |
| - Study title - Study author - Study year - Country of origin - Study type - Sample size of intervention group - Sample size of comparison group - Comparison group type   - Control   - Optimal medical therapy - Method of injection   - Intracoronary   - Intravenous,   - Intramuscular   - Intracoronary/intravenous - Type of cell injected   - Bone marrow mesenchymal stem cells   - Wharton’s jelly mesenchymal stem cells   - Unspecified mesenchymal stem cells   - Bone marrow progenitor cells   - Unspecified progenitor cells - Source of cell   - Bone marrow   - Wharton’s jelly   - Umbilical cord   - Circulation   - Manufactured | - LVEF in therapy group   - Pretreatment   - Posttreatment   - Improvement - LVEF in control group   - Pretreatment   - Posttreatment   - Improvement - Imaging modality for LVEF - Time between pretreatment and posttreatment measurement of LVEF - ESV in therapy group   - Pretreatment   - Posttreatment   - Improvement - ESV in control group   - Pretreatment   - Posttreatment   - Improvement - EDV in therapy group   - Pretreatment   - Posttreatment   - Improvement - EDV in control group   - Pretreatment   - Posttreatment   - Improvement - Imaging modality for ESV/EDV - Time between pretreatment and posttreatment measurement of ESV/EDV - # of patients experiencing grouped MACE in therapy group - # of patients experiencing individual MACE in therapy group   - Death   - Recurrent MI   - Readmission for heart failure   - Unspecified hospitalization   - Revascularization of target vessel   - Revascularization of nontarget vessel   - Unspecified vessel revascularization   - Stent thrombosis   - Stroke   - Myocardial fibrosis   - Microvascular embolization   - Ectopic tissue formation - # of patients experiencing grouped MACE in control group - # of patients experiencing individual MACE in control group   - Death   - Recurrent MI   - Readmission for heart failure   - Unspecified hospitalization   - Revascularization of target vessel   - Revascularization of nontarget vessel   - Unspecified vessel revascularization   - Stent thrombosis   - Stroke   - Myocardial fibrosis   - Microvascular embolization   - Ectopic tissue formation - Time interval until measurement of MACE | - Myocardial viability in therapy group   - Pretreatment   - Posttreatment   - Improvement - Myocardial viability in control group   - Pretreatment   - Posttreatment   - Improvement - Imaging modality for myocardial viability - Time between pretreatment and posttreatment measurement of myocardial viability - Myocardial perfusion defect in therapy group   - Pretreatment   - Posttreatment   - Improvement - Myocardial perfusion defect in control group   - Pretreatment   - Posttreatment   - Improvement - Time between pretreatment and posttreatment measurement of myocardial perfusion defect - CFR in therapy group   - Pretreatment   - Posttreatment   - Improvement - CFR in control group   - Pretreatment   - Posttreatment   - Improvement - Time between pretreatment and posttreatment measurement of CFR - Adenosine-induced MVRI in therapy group   - Pretreatment   - Posttreatment   - Improvement - Adenosine-induced MVRI in control group   - Pretreatment   - Posttreatment   - Improvement - Time between pretreatment and posttreatment measurement of adenosine-induced MVRI - SV in therapy group   - Pretreatment   - Posttreatment   - Improvement - SV in control group   - Pretreatment   - Posttreatment   - Improvement - Imaging modality for SV - Time between pretreatment and posttreatment measurement of SV |

Table 1. Data extraction elements for both systematic review and meta-analysis.
*Footnote: Analysis contingent on sufficient data availability
