# Supplementary material for: Mesenchymal Stem Cell Therapy for Acute Myocardial Infarction: Protocol for a Systematic Review and Meta-Analysis
Source: JMIR Res Protoc. 2025 Feb 6;14:e60591. doi: 10.2196/60591 (PMC11843057; doi:10.2196/60591)
Supplement: Multimedia Appendix 4 [file resprot_v14i1e60591_app4.docx]

**Textbox S1.**

| Quality assessment of controlled intervention studies  **Criteria**   - Was the study described as randomized, a randomized trial, a randomized clinical trial, or an RCT? - Was the method of randomization adequate (ie, use of randomly generated assignment)? - Was the treatment allocation concealed (so that assignments could not be predicted)? - Were study participants and providers blinded to treatment group assignment? - Were the people assessing the outcomes blinded to the participants' group assignments? - Were the groups similar at baseline on important characteristics that could affect outcomes (eg, demographics, risk factors, and comorbid conditions)? - Was the overall drop-out rate from the study at endpoint 20% or lower of the number allocated to treatment? - Was the differential drop-out rate (between treatment groups) at endpoint 15 percentage points or lower? - Was there high adherence to the intervention protocols for each treatment group? - Were other interventions avoided or similar in the groups (eg, similar background treatments)? - Were outcomes assessed using valid and reliable measures, implemented consistently across all study participants? - Did the authors report that the sample size was sufficiently large to be able to detect a difference in the main outcome between groups with at least 80% power? - Were outcomes reported or subgroups analyzed prespecified (ie, identified before analyses were conducted)? - Were all randomized participants analyzed in the group to which they were originally assigned, that is, did they use an intention-to-treat analysis? |
| --- |
